# An Increased Plasma Level of ApoCIII-Rich Electronegative High-Density Lipoprotein May Contribute to Cognitive Impairment in Alzheimer’s Disease

**DOI:** 10.3390/biomedicines8120542

**Published:** 2020-11-26

**Authors:** Hua-Chen Chan, Liang-Yin Ke, Hsiao-Ting Lu, Shih-Feng Weng, Hsiu-Chuan Chan, Shi-Hui Law, I-Ling Lin, Chuan-Fa Chang, Ye-Hsu Lu, Chu-Huang Chen, Chih-Sheng Chu

**Affiliations:** 1 Center for Lipid Biosciences, Kaohsiung Medical University Hospital, Kaohsiung Medical University, Kaohsiung 807377, Taiwan; huachen.chan@gmail.com (H.-C.C.); kly@gap.kmu.edu.tw (L.-Y.K.); earth1981709@hotmail.com (H.-C.C.); yehslu@cc.kmu.edu.tw (Y.-H.L.); 2 Department of Medical Laboratory Science and Biotechnology, College of Health Sciences, Kaohsiung Medical University, Kaohsiung 807378, Taiwan; b430502@gmail.com (H.-T.L.); shlaw_0909@hotmail.com (S.-H.L.); linili@kmu.edu.tw (I.-L.L.); affa@mail.ncku.edu.tw (C.-F.C.); 3 Graduate Institute of Medicine, College of Medicine & Drug Development and Value Creation Research Center, Kaohsiung Medical University, Kaohsiung 807378, Taiwan; 4 Department of Healthcare Administration and Medical Informatics, College of Health Sciences, Kaohsiung Medical University, Kaohsiung 807378, Taiwan; sfweng@kmu.edu.tw; 5 Department of Medical Laboratory Science and Biotechnology, College of Medicine, National Cheng Kung University, Tainan 701401, Taiwan; 6 Division of Cardiology, Department of International Medicine, Kaohsiung Medical University Hospital, Kaohsiung 807377, Taiwan; 7 Vascular and Medicinal Research, Texas Heart Institute, Houston, TX 77030, USA; cchen@texasheart.org; 8 Division of Cardiology, Department of Internal Medicine, Kaohsiung Municipal Ta-Tung Hospital, Kaohsiung 80145, Taiwan

**Keywords:** high-density lipoprotein (HDL), Alzheimer’s disease (AD), cognitive impairment

## Abstract

High-density lipoprotein (HDL) plays a vital role in lipid metabolism and anti-inflammatory activities; a dysfunctional HDL impairs cholesterol efflux pathways. To understand HDL’s role in patients with Alzheimer’s disease (AD), we analyzed the chemical properties and function. HDL from AD patients (AD-HDL) was separated into five subfractions, H1–H5, using fast-protein liquid chromatography equipped with an anion-exchange column. Subfraction H5, defined as the most electronegative HDL, was increased 5.5-fold in AD-HDL (23.48 ± 17.83%) in comparison with the control HDL (4.24 ± 3.22%). By liquid chromatography mass spectrometry (LC/MS^E^), AD-HDL showed that the level of apolipoprotein (apo)CIII was elevated but sphingosine-1-phosphate (S1P)-associated apoM and anti-oxidative paraoxonase 1 (PON1) were reduced. AD-HDL showed a lower cholesterol efflux capacity that was associated with the post-translational oxidation of apoAI. Exposure of murine macrophage cell line, RAW 264.7, to AD-HDL induced a vibrant expression of ganglioside GM1 in colocalization with apoCIII on lipid rafts alongside a concomitant increase of tumor necrosis factor-α (TNF-α) detectable in the cultured medium. In conclusion, AD-HDL had a higher proportion of H5, an apoCIII-rich electronegative HDL subfraction. The associated increase in pro-inflammatory (apoCIII, TNF-α) components might favor Amyloid β assembly and neural inflammation. A compromised cholesterol efflux capacity of AD-HDL may also contribute to cognitive impairment.

## 1. Introduction

Alzheimer’s disease (AD) is a chronic neurodegenerative disease affecting more than 35 million people worldwide [1]. AD patients’ brains show irreversible loss of neurons and atrophy, accumulating senile plaques and neurofibrillary tangles [2,3,4]. Due to the proteolytic activity of β-secretase (BACE), amyloid precursor protein (APP) can be cleaved into Amyloid Aβ_42_ [5]. The other identifiable feature is hyper-phosphorylated and abnormally folded Tau protein within the neurons [6]. Apart from these clinically distinct features, many reports show that lipid metabolism dysregulation is associated with Alzheimer’s disease [7,8,9,10,11]. Ceramide and lysophosphatidylcholine (LPC) were particularly identified in the pathogenesis of AD [12,13,14,15,16]. Ceramide regulates the molecular stability of BACE to promote Aβ_42_ overproduction, which enhances sphingomyelinase activity to overproduce ceramide. In contrast, decreasing ceramide production by deleting neutral sphingomyelinase-2 (nSMase2), as generated in nSMase2-deficient 5XFAD mice models (fro;5XFAD), improves pathology and improves cognition status [17]. Likewise, LPC induces pericyte loss, endothelial leakage, the downregulation of junction proteins (claudin-5, zonula occludens-1) and neuronal demyelination [15]. Treatment with a prostacyclin analog, a vasodilator and platelet aggregation inhibitor attenuated LPC-mediated vascular dysfunction in the adult mouse spinal cord [18].

The contribution of lipids and apolipoproteins in AD development has been the subject of research interests [19,20,21]. In the bloodstream, lipoproteins are the primary carriers for lipids and are responsible for lipid metabolism. Triglycerides and cholesteryl ester are encapsulated in the core of lipoproteins, while phospholipid, cholesterol and apolipoproteins are cladding outside to stabilize the structure of the lipoproteins [22]. High-density lipoprotein (HDL) plays an essential role in lipid metabolism and anti-inflammatory activities [23,24]. The reverse cholesterol transfer (RCT) function of HDL can be relatively quantified in vitro by removing fluorescent cholesterol from macrophages [25,26]. In contrast, dysfunctional HDL impairs the ATP-binding cassette sub-family A member 1 (ABCA1) or scavenger receptor class B member 1 (SRB1)-mediated cholesterol efflux pathways [27]. The dysfunctional HDL is characterized as apoAI oxidation [28,29] and other shreds of evidence show that apoAI plays a protective role in avoiding Aβ aggregation [30]. *ApoAI* gene deficiency causes cognitive impairment in mice; in contrast, overexpressing apoAI shows protective effects [31]. As previous studies classified electronegative HDL as the dysfunctional subfraction [30,31], in this study, we aimed to analyze the chemical properties of electronegative HDL from AD patients or healthy (control) subjects. 

## 2. Materials and Methods

### 2.1. Study Participants

This study was an extension study of the brain research carried out at Kaohsiung Medical University. The relevant clinical information will be published but not in this basic research. All procedures were approved by the institutional review board of Kaohsiung Medical University Hospital (KMUH), Kaohsiung, Taiwan, KMUHIRB-SV(I)-20150008, approval date 10 April 2015. A total of 44 patients and 24 healthy control cases were enrolled in this project. All participants gave written consent as per the Declaration of Helsinki. The diagnosis of AD in all participants was made by clinical neurologists in KMUH according to the 2011 National Institute of Neurological and Communicative Disorders and Stroke (NINCDS) and the Alzheimer’s Disease and Related Disorders Association (ADRDA) workgroup [32]. Due to the difficulty of recruiting age-matched healthy controls, this project had limitations and therefore focused on examining HDL’s quality. For all study participants, biochemical measurements were performed at the Department of Laboratory Medicine at KMUH (accredited by Taiwan Accreditation Foundation).

### 2.2. HDL Mobility, Components and Subfractionation

Blood samples were anti-coagulated with ethylenediaminetetraacetic acid (EDTA) and centrifuged at 3000 rpm for 10 min (4 °C, Allegra^®^ X-12R Centrifuge; Beckman Coulter; CA, USA). Plasma samples were collected and treated with cOmplete^TM^ Protease Inhibitor (Roche; Basel, Switzerland) and ethylenediaminetetraacetic acid (EDTA; Scharlau; Spain). HDL samples were isolated from plasma using sequential ultracentrifugation (Type 90Ti; Beckman Coulter; IN, USA) [33,34]. The HDL samples were further dialyzed against buffer A (1 M Tris buffer, pH 8.0 and 0.5M EDTA) for 24 h for three times.

Agarose gel (0.75%) electrophoresis was used to determine the electronegativity of the total HDL. Relative mobility was quantified by dividing the distance the sample band migrated by the distance the dye front migrated [35]. HDL components were analyzed with Enzychorom^TM^ Assay kits for triglyceride, phospholipid, total cholesterol and cholesteryl ester (Bioassay Systems; CA, USA), according to the manufacturer’s instructions. An equal amount of HDL samples was further injected into the ÄKTA fast-protein liquid chromatography system (GE Healthcare Life Sciences) equipped with UnoQ12 anion-exchange columns (Bio-Rad Laboratories). HDL can be divided into five subfractions (H1–H5) by using a salt gradient for elution. The HDL subfractions were concentrated by using Centriprep filters (YM-30; EMD Millipore) and sterilized by passing them through a 0.22 μm filter [36].

### 2.3. Lipid Extraction

Lipids were extracted from HDL samples of AD patients or controls; protocols were the same as previously described [37,38]. In short, 30 μg of HDL was transferred to a glass tube and 1 mL of H_2_O (EMD Millipore; MA, USA), 2.5 mL of methanol (Fisher Scientific; MA, USA) and 1.25 mL of CHCl_3_ (Macron fine chemicals, Fisher Scientific; MA, USA) were added to the HDL samples and vortexed for 15 s. Another 0.9 mL of H_2_O and 1.25 mL of CHCl_3_ were then added and vortexed. After centrifugation at 3000 rpm for 10 min, we extracted the bottom layer of organic solvents and transferred them into a 12 × 32 mm glass vial (Waters Corporation; MA, USA) and dried with nitrogen gas (Nitrogen generator; Anest Iwata; Japan) on a thermo module (Eyela; Japan). The dried pellets were dissolved in 0.25 mL of a sample solution containing isopropanol (EMD Millipore; MA, USA), acetonitrile (EMD Millipore; MA, USA) and H_2_O in a 2:1:1 ratio.

### 2.4. Liquid Chromatography Mass Spectrometry for Lipid Analysis

HDL-lipid extracts were separated on an Acquity® ultra-performance liquid chromatography (UPLC; Waters Corporation; MA, USA) equipped with a CSH^TM^ C-18 column under gradient conditions at a flow rate of 100 uL/min over 20 min at 55 °C. The mobile phase A was composed of 10 mM NH_4_HCO_2_ in ACN/H_2_O (60/40) and 0.1% formic acid (0.1% *v*/*v*, EMD Millipore; MA, USA); mobile phase B was composed of 10mM NH_4_HCO_2_ in IPA/ACN (90/10) and 0.1% formic acid (0.1% *v*/*v*) for molecule protonation. Mass spectrometry was performed on a XEVO Q-tof G2 (Waters Corporation; MA, USA) instrument equipped with an electrospray ionization interface (ESI) and operated in the data-independent collection mode (MS^E^). The cone voltage was 30 kV. Parallel ion fragmentation was programmed to switch between low (4 eV) and high (35–55 eV) energies in the collision cell. Signals were collected from 250 to 1600 m/z utilizing leucine enkephalin as the lock mass calibrant (m/z = 556.2771 Da; Waters Corporation, MA, USA). Data were processed with MarkerLynx (Waters Corporation, MA, USA) and Progenesis QI software (Nonlinear Dynamics, Waters Corporation, MA, USA).

### 2.5. Protein Quantification and Peptide Digestion

A Pierce^TM^ BCA Protein Assay Kit (Thermo Fisher Scientific; MA, USA) was used to quantify HDL samples. A total of 30 μg of each HDL sample and 50 fmol of yeast alcohol dehydrogenase (ADH; the protein quantification control) were incubated with 100 uL RapiGest SF (Waters Corporation, MA, USA) and 5 μL DTT (Dithiothreitol; Sigma, Canada) at 60 °C for 30 min. Later, 20 μL IAM (iodoacetamide; Sigma, USA) was added and then incubated in the dark at room temperature for another 30 min. Samples were then transferred to an Amicon® Ultra Centrifugal Filter (EMD Millipore; MA, USA) and centrifuged at 4 °C and 14,000 rpm for 5 min. After washing six times with 450 μL ammonium bicarbonate (Sigma-Aldrich, Spain), 3 μL trypsin gold (0.1%; mass spectrometry grade; Promega, USA) was added and incubated at 37 °C overnight. The reaction was stopped by 1 μL formic acid. After being centrifuged at 13,000 rpm for 10 min, the supernatant was transferred to a 12 × 32 mm glass vial (Waters Corporation; MA, USA) for mass spectrometry.

### 2.6. M-class UPLC and Mass Spectrometry for Protein Analysis

The protein composition of HDL samples was identified and quantified by the use of a Waters Xevo G2 mass spectrometer (Waters Corporation; MA, USA). In brief, the peptide samples were chromatographically separated on an M-class ultra-performance liquid chromatography (UPLC; Waters Corporation; MA, USA) equipped with an ACQUITY UPLC BEH C18 Column (130 Å, 1.7 µm, 2.1 mm × 50 mm) under gradient conditions at a flow rate of 300 nL/min over 60 min at 35 °C. The mobile phase was composed of acetonitrile as the organic modifier and formic acid (0.1% *v*/*v*) for molecule protonation. Peptide fragmentation was performed on a high-definition mass spectrometer (HDMS) instrument equipped with a nano-electrospray ionization (nano-ESI) and operated in the MS^E^ mode. Parallel ion fragmentation was programmed to switch between low (4 eV) and high (15–45 eV) energies in the collision cell and data was collected from 300 to 3500 m/z utilizing glu-fibrinopeptide B as the separate data channel lock mass calibrant (m/z = 785.8426 Da; Waters Corporation; MA, USA) [39]. Data were processed with ProteinLynx GlobalServer v3.0 (Waters Corporation; MA, USA) [40] and Progenesis QI for proteomics (Nonlinear Dynamics, Waters Corporation; MA, USA). Deisotoped results were searched for protein association from the Uniprot (www.uniprot.org) human protein database [41,42].

### 2.7. Cell Culture

Murine macrophage cell line, RAW 264.7 cells, was purchased from the Bioresource Collection and Research Center (BCRC; Hsin-Chu, Taiwan). A range of 5–10 passage cells were cultured in Dulbecco’s modified Eagle medium (DMEM, Invitrogen; Massachusetts, USA) supplemented with 10% fetal bovine serum (FBS) (Gibco Laboratory; Massachusetts, USA), 100 U/mL penicillin (Invitrogen; Massachusetts, USA) and 100 U/mL streptomycin (Invitrogen; MA, USA) in a humidified atmosphere of 5% CO_2_ at 37 °C.

### 2.8. Cholesterol Efflux Assay

RAW 264.7 cells were incubated with 5 μg/mL 22-NBD-cholesterol (22-(N-(7-Nitrobenz-2-Oxa-1,3-Diazol-4-yl)Amino)-23,24-Bisnor-5-Cholen-3β-ol (Invitrogen; MA, USA). After 24 h, cells were washed and maintained in DMEM and 0.3 mmoL/L cyclic adenosine 3’,5’-monophosphate (cAMP; Roche Applied Science, Penzberg, Switzerland) for another 12 h. Cells were then treated with 0, 25, 50, 75 or 100 µg/mL of HDL, either isolated from controls or from AD patients.

### 2.9. Sodium Dodecyl Sulfate-Polyacrylamide Gel (SDS-PAGE) and Western Blot Testing

Cells were lysed on ice with a radio-immunoprecipitation assay (RIPA) buffer (VWR; Radnor, PA) and a protease inhibitor cocktail (Roche Diagnostics, Indianapolis, IN, USA). Cell lysates were centrifuged at 20,000× *g* for 20 min at 4 °C. Next, 20 μg of supernatants were transferred to sodium dodecyl sulfate-polyacrylamide gel (SDS-PAGE) for electrophoresis. Proteins in the polyacrylamide gel were transferred to a polyvinylidene difluoride (PVDF) membrane (Millipore, Temecula, CA, USA). Membranes were incubated in 50 g/L non-fat milk at 4 °C overnight to block non-specific bindings and were then incubated with primary antibodies. The primary antibodies were ABCA1 (1:100, GeneTex, California, USA), a lectin-type oxidized LDL receptor (LOX-1) (1:1000, Origene, Rockville, USA), phosphor-NF-κB p65 (1:1000, GeneTex) and β-actin (1:5000, Sigma-Aldrich). The secondary antibodies were an HRP-linked rabbit IgG antibody (1:1000, GeneTex) or an HRP-linked mouse IgG antibody (1:1000, GeneTex). Membranes were incubated with secondary peroxidase-conjugated anti-goat, anti-rabbit or anti-mouse IgG at room temperature for one hour. The results were detected by using an enhanced chemiluminescence kit (Millipore, Temecula, CA) and were quantitated with Fusion Solo S (Vilber Lourmat, Eberhardzell, Germany).

### 2.10. Enzyme Linked Immunosorbent Assay (ELISA)

Tumor necrosis factor (TNF)-α ELISA kits were purchased from R&D Systems (Minneapolis, MN, USA). All procedures of detection were done according to the manufacturer’s instructions.

### 2.11. Immunofluorescence Staining

RAW cells were treated with phosphate-buffered saline (PBS), control HDL (Ctl-HDL), AD patients’ HDL (AD-HDL), control H1 HDL (H1) or AD patients’ H5 HDL (H5). After 2 h, plasma GM1 ganglioside on cells were labeled following the manufacturer’s instructions of the Alexa Fluor 555 lipid raft labeling kit (Thermo Fisher Scientific; MA, USA). In brief, cells were pelleted and resuspended in the fluorescent CT-B conjugated working solution for 10 min at 4 °C. After incubation, we gently washed the cells three times with chilled 1X PBS. We then incubated the cells with chilled anti-CT-B antibody for 30 min at 4 °C. Labeled cells were fixed in chilled 1X PBS containing 4% formaldehyde for 15 min at 4 °C and permeabilized with 0.1% triton X-100 in 1X PBS. Cells were then labeled with the anti-apolipoprotein CIII (apoCIII) antibody (Academy Biomedical Company; TX, USA) and the Alexa Fluor 488-conjugated rabbit secondary antibody. Images from similar regions of each section were captured by using a confocal laser scanning microscope (Zeiss LSM 700).

### 2.12. Statistical Analysis

Data were expressed as means ± standard deviation (SD) and media (interquartile range; IQR). Statistics were done by using SPSS Statistics v20 (IBM, NY) and Prism 5 (GraphPad Software, Inc., San Diego, CA). The significance of the difference between the two groups was determined by a two-sample t-test, Wilcoxon rank-sum test or a chi-squared test. Comparisons among three more groups were determined by one-way analysis of variance (ANOVA) followed by the nonparametric Kruskal–Wallis test. A value of *P* < 0.05 was considered to be significant.

## 3. Results

### 3.1. The HDL from AD Patients Show Different Components 

The biochemical profiles of the 68 subjects are listed in Table 1 and categorized into two groups, Alzheimer’s patients (AD; *n* = 44) and healthy controls (controls; *n* = 24). There was no significant change in the concentration of glutamic-pyruvate transaminase (GPT), indicating that the liver function of the AD group was not different. The level of triglyceride was similar between the two groups. The total cholesterol (T-CHOL), HDL-C and LDL-C were relatively decreased in the AD group but within the reference range. 

**Figure 1:**
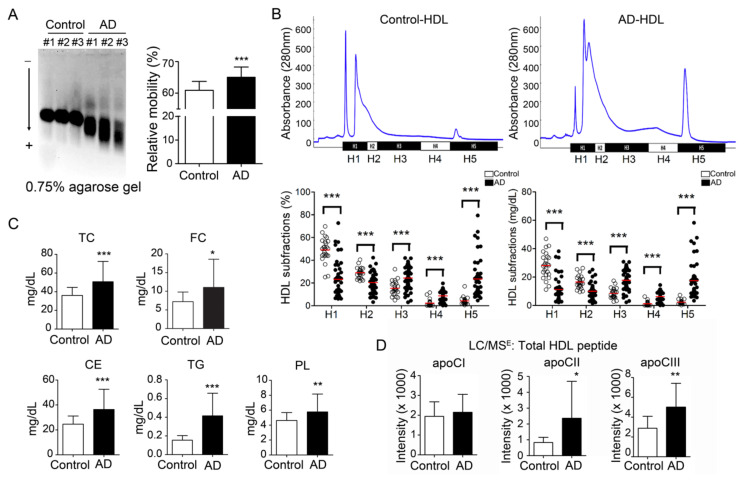
Electronegativity of high-density lipoprotein (HDL) was increased in Alzheimer’s disease (AD) patients. (**A**) HDL from AD patients show higher relative mobility than controls. (**B**) Representative patterns of HDL subfractionation by using fast-protein liquid chromatography. The lower panel shows that the percentage and quantity of H5 HDL were elevated in AD patients. Red line: mean value. (**C**) By using a colorimetric method, lipids were quantified. The lipid components were increased in HDL from AD patients. (**D**) Apolipoproteins were quantified by mass spectrometry. Results showed that apoCII and apoCIII were all increased. * *p* < 0.05, ** *p* < 0.01, *** *p* < 0.001, compared with controls and AD patients. Abbreviations. H1–H5: the five HDL subfractions divided according to electronegativity; TC: total cholesterol; FC: free cholesterol; CE: cholesteryl ester; TG: triglyceride; PL: phospholipids; ApoCI: apolipoprotein CI; ApoCII: apolipoprotein CII; ApoCIII: apolipoprotein CIIII.

For the HDL components, the total protein was significantly decreased in the HDL of AD patients (68.7 ± 13.2%) in comparison with the healthy controls (77.3 ± 5.2%; *p* < 0.001) (Table 1). Triglyceride (TG; 0.21 ± 0.12% vs. control 0.08 ± 0.03%; *p* < 0.0001), phospholipids (PL; 5.77 ± 2.39% vs. control 4.63 ± 1.05%; *p* < 0.01), free cholesterol (FC; 7.12 ± 3.14% vs. control 5.69 ± 1.26%; *p* = 0.01) and cholesteryl ester (18.2 ± 8.12% vs. control 12.31 ± 3.27%; *p* < 0.0001) were all increased in HDL from AD patients. (Figure 1C).

### 3.2. Electronegativity of HDL was increased in AD patients

By electrophoresis on 0.75% agarose gel, the HDL from AD patients showed higher relative mobility than HDL from healthy controls (65.0 ± 3.2% vs. control 61.1 ± 2.7%; *p* < 0.001) (Figure 1A). Furthermore, we divided HDL into five increasingly electronegative subfractions by using liquid chromatography equipped with an anion-exchange column. The percentage of H1 was decreased in the total HDL from AD patients (23.4 ± 16.3% vs. control 48.8 ± 10.5%; *p* < 0.001) (Figure 1B, lower left panel). In contrast, the percentage of H5 was higher in the total HDL from AD patients (23.5 ± 18.0% vs. control 4.2 ± 3.3%; *p* < 0.001). While calculating the concentration, H1 and H2 HDL were decreased in AD patients (H1: 11.5 ± 9.0 mg/dL vs. control 28.1 ± 9.1 mg/dL; *p* <0.001; H2: 10.2 ± 5.4 mg/dL vs. control 16.7 ± 4.4 mg/dL; *P* < 0.01) (Figure 1B, lower right panel). In contrast, H5 HDL was elevated in AD patients (10.8 ± 9.1 mg/dL vs. control 2.3 ± 1.6 mg/dL; *p* < 0.001).

### 3.3. Apolipoprotein CII and CIII Were Increased in HDL from AD Patients

By M-class UPLC/MS^E^ equipment and Progenesis QI-P software, the protein components were relatively quantified. The results showed that apoCII and apoCIII were significantly higher in the HDL from AD patients (apoCII: 2358 ± 2330 vs. control 829 ± 327, *P* < 0.05; apoCIII: 4999 ± 2413 vs. control 2878 ± 1209; *p* < 0.01) (Figure 1D).

### 3.4. HDL from AD Patients Show Impaired Function in Cholesterol Efflux

**Figure 2:**
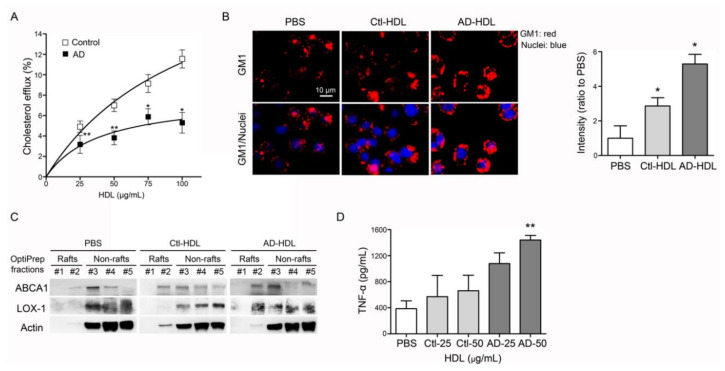
Analysis of function and proinflammatory properties of HDL. (**A**) Function of reverse cholesterol efflux was decreased in HDL from AD patients. (**B**) AD-HDL induced vibrant expression of ganglioside GM1 on the membrane of marine macrophages, RAW 264.7 cells. AD-HDL enhanced the higher expression levels of (**C**) LOX-1 on the membrane and (**D**) TNF-α in the conditioned culture media. * *p* < 0.05, ** *p* < 0.01. Abbreviations. AD: Alzheimer’s disease; PBS: phosphate-buffered saline; Ctl-HDL: HDL from controls; AD-HDL: HDL from AD patients; ABCA1: ATP-binding cassette, sub-family A member 1; LOX-1: lectin-type oxidized LDL receptor; TNF-α: tumor necrosis factor-α.

Murine RAW 264.7 macrophages were treated with 22-NBD-cholesterol and HDL either from controls or AD patients. Conditioned culture media were collected and the fluorescence signals were quantified. While treated with control HDL, the Vmax was 23.4 ± 7.3 and Km 108.9 ± 57.0 (*n* = 22). In contrast, the Vmax was 7.9 ± 2.7 and Km 39.9 ± 35.5 (*n* = 17) when treated with HDL from AD patients (Figure 2A). These results indicate that the function of reverse cholesterol efflux was decreased in HDL from AD patients.

### 3.5. HDL from AD Patients Show Proinflammatory Properties

Forty minutes’ exposure of RAW 264.7 cells to HDL from AD patients (AD-HDL) induced a vibrant expression of ganglioside GM1 (Figure 2B). By using OptiPrep density centrifugation, lipid raft fractions were isolated from RAW 264.7 cells treated with AD-HDL to observe the changing properties of the membranes. The results from Western Blot testing showed that ABCA1 and LOX-1 were translocated on to the cell membrane while RAW 264.7 cells were treated with AD-HDL (Figure 2C). The inflammatory cytokines were evaluated by ELISA. The results indicated that 50 µg/mL of AD-HDL induced higher expression levels of tumor necrosis factor-α (TNF-α) from RAW 264.7 cells (*n* = 4, *p* < 0.01) (Figure 2D).

### 3.6. The Functional Impairment of AD-H1 and the Proinflammatory Properties of AD-H5

**Figure 3:**
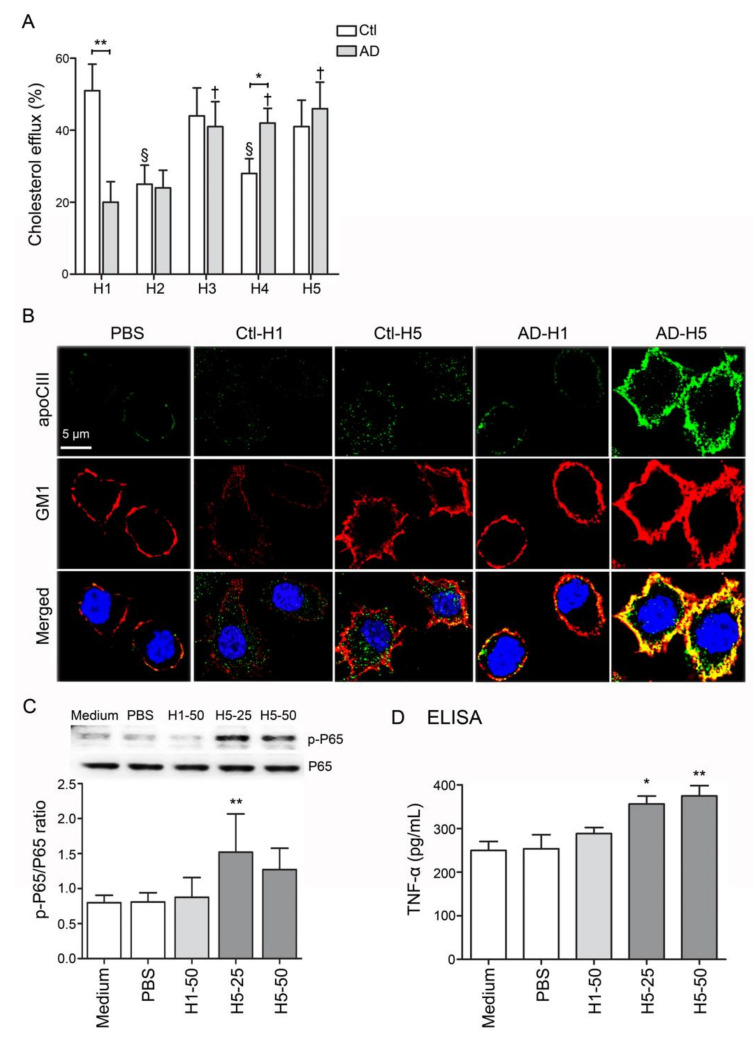
Analysis of function and proinflammatory properties of HDL subfractions. (**A**) RCT function was reduced in comparison with H1 HDL from healthy subjects. (**B**) AD-H5 induced a higher expression level of GM1 on the membrane and co-localized with APOCIII. (**C**, **D**) AD-H5 HDL induced p-P65 and TNF-α overexpression in RAW 264.7 cells. * *p* < 0.05, ** *p* < 0.01. Abbreviations. AD: Alzheimer’s disease; Ctl: controls; PBS: phosphate-buffered saline; apoCIII: apolipoprotein CIII; GM1: ganglioside GM1; TNF-α: tumor necrosis factor-α.

In addition to the decreased levels of H1 HDL, the function of reverse cholesterol transport (RCT) was also significantly impaired in H1 HDL from AD patients (AD-H1; *n* = 6, *p* < 0.01; Figure 3A). Other than this, the RCT function was relatively decreased in H2 to H5 subfractions in comparison with H1 from healthy subjects (Ctl-H1; Figure 3A). While RAW 264.7 cells were treated with AD-H5 for forty minutes, the expression level of GM1 on the membrane was elevated and colocalized with apoCIII (Figure 3B). Additionally, AD-H5 HDL may induce higher expression levels of nuclear factor-kappa-B p65 subunit (p-P65) and TNF-α, indicating the proinflammatory properties to RAW 264.7 cells (Figure 3C,D).

### 3.7. The Post-Translational Modifications of HDL

**Figure 4:**
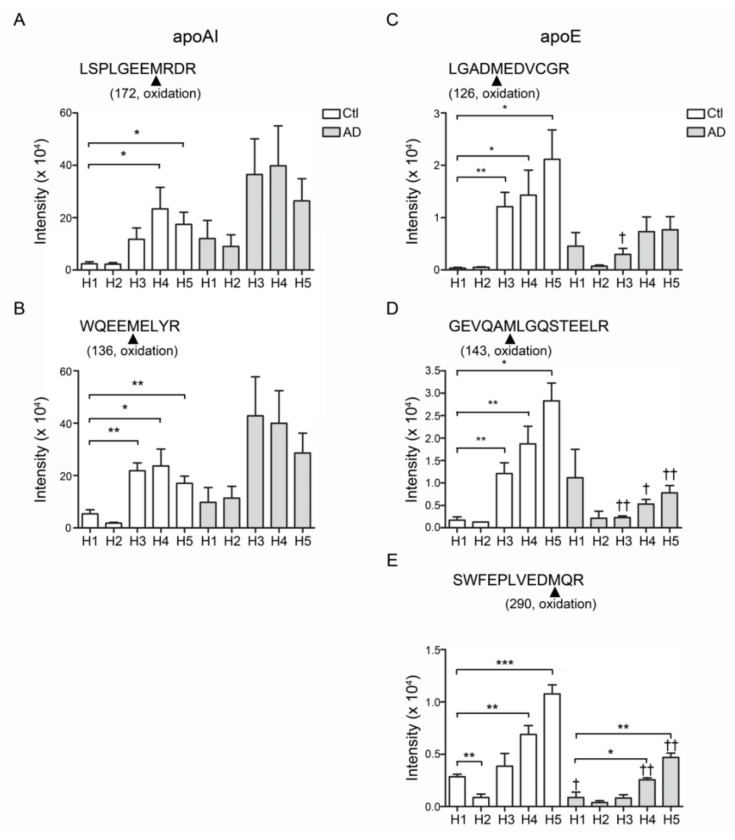
The post-translational modification of apoA1 and apoE in HDL. The oxidation was found and increased on the position 136 and 172 methionine (136M, 172M) of apoAI (**A**) peptide sequences WQEEM*ELYR and (**B**) LSPLGEEM*RDR. The position 126, 143 and 290 methionine (126M, 143M, 290M) of apoE was also oxidized. (**C**) peptide sequences LGADM*EDVCGR. (**D**) GEVQAM*LGQSTEELR. (**E**) SWFEPLVEDM*QR. * *p* < 0.05, ** *p* < 0.01, *** *p* < 0.001.

Compared with control-H1, the peptide fragments of apoAI and apoE showed higher levels of oxidation in H3, H4 and H5 from healthy controls or from AD patients (Figure 4). The oxidation was detected on the position 136 and 172 methionine (136M, 172M) of apoAI (peptide sequences WQEEM*ELYR and LSPLGEEM*RDR; Figure 4A,B) and the position 126, 143 and 290 methionine (126M, 143M, 290M) of apoE (peptide sequences LGADM*EDVCGR, GEVQAM*LGQSTEELR and SWFEPLVEDM*QR; Figure 4C–E). In addition to these, the AD-H1 also showed higher levels of APOA-I 136M, 172M and apoE 126M, 143M oxidation (Figure 4A–D).

### 3.8. Lipidomic Study of HDL from AD Patients and Controls

**Figure 5:**
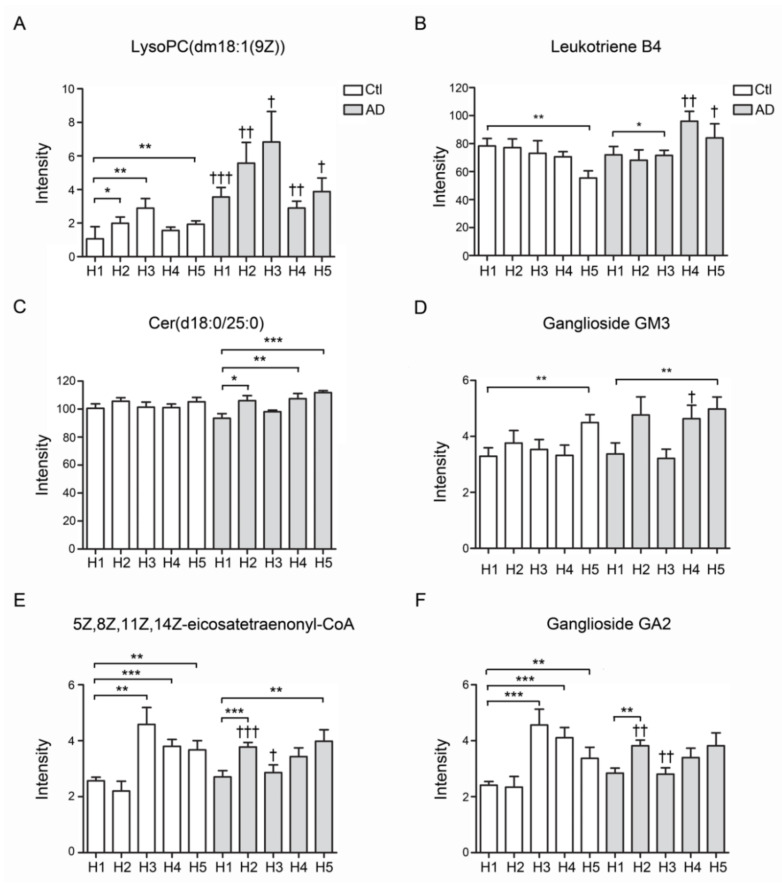
Lipidomics analysis of HDL form AD patients and controls. (**A**) LPC (dm18:1(9Z)) was significantly increased in all subfractions of AD-HDL in comparison with Ctl-HDL. (**B**) Eukotriene was significantly decreased in the H5 of Ctl-HDL. (**C**) Cer(d18:0/25:0) ceramide was elevated in H4 and H5 of AD-HDL in comparison with H1. (**D**) Ganglioside GM3 was elevated in the H5 of AD-HDL and Ctl-HDL subfractions. (**E**) Eicosatetraenonyl-CoA was elevated in H3–H5 from Ctl-HDL and H2 and H5 from AD-HDL. (**F**) Ganglioside GA2 was enhanced in H3–H5 from Ctl-HDL and H2 and H5 from AD-HDL. * *p* < 0.05, ** *p* < 0.01, *** *p* < 0.001: comparison to H1 of either AD patients or controls; † *p* < 0.05, †† *p* < 0.01, ††† *p* < 0.001: comparison between AD patients and controls.

The total lipids were extracted from all subfractions of AD patients and controls. Lipid contents were quantified and analyzed by Progenesis QI software. The results showed that the LysoPC (dm18:1(9Z)) was significantly increased in all subfractions of AD-HDL in comparison with Ctl-HDL (Figure 5A). Leukotriene was elevated in the subfractions of H4 and H5 of AD-HDL (*p* < 0.01 and *p* < 0.05). In contrast, leukotriene was significantly decreased in the H5 of Ctl-HDL (Figure 5B). For the Cer(d18:0/25:0) ceramide content, there was no change in the control HDL subfractions but it was elevated in H4 and H5 of AD-HDL in comparison with H1 (Figure 5C; *p* < 0.01 and *p* < 0.001, respectively). Regarding ganglioside GM3, it was elevated in the H5 of AD-HDL and Ctl-HDL subfractions (Figure 5D; *p* < 0.01). Compared with H1 from Ctl-HDL, eicosatetraenonyl-CoA was elevated in H3–H5 from Ctl-HDL and H2 and H5 from AD-HDL (Figure 5E; *p* < 0.001). Finally, the ganglioside GA2 was enhanced in H3–H5 from Ctl-HDL and H2 and H5 from AD-HDL (Figure 5F).

## 4. Discussion

Previous studies have demonstrated that electronegative lipoproteins such as electronegative LDL (L5) or electronegative VLDL (V5) are pro-atherogenic and pro-thrombotic, leading to endothelial dysfunction and vascular inflammation [33,38,43,44,45,46,47]. Notably, L5 and V5 showed regulatory effects on glial cells and contributed to neurodegenerative disorders such as AD [48,49]. In the current research, we demonstrated that the electronegativity of AD-HDL was increased (Figure 1A,B). The RCT function of HDL was impaired (Figure 2A) and was prone to be pro-inflammatory on immune cells (Figure 2B,D). The H1 HDL of AD patients was dysfunctional (Figure 3A); H5 HDL induced GM3 ganglioside overexpression on the membrane of macrophages (Figure 3B) and enhanced inflammatory cytokines p-P65 and TNF-α in a dose-dependent manner (Figure 3C,D).

According to a proteome analysis, the composition of HDL particles is dynamic [50]; for instance, the protein/lipid ratio is decreased during HDL’s maturation [51]. ApoE and PON1 are incorporated into HDL to exert cholesterol clearance and antioxidation [50]. In addition, the HDL protein contents are altered in patients with atherosclerosis [52,53], diabetes [54], coronary artery disease [54,55] and non-alcoholic fatty liver disease [56]. In this study, the ratio of protein to lipid content was lower in HDL from AD patients (Table 1). The RCT function of HDL from AD patients was also decreased (Figure 2A and Figure 3A). On the contrary, the lipid contents including triglyceride, phospholipids, free cholesterol and cholesteryl esters were all increased (Table 1). The reduction of the protein/lipid ratio and the increase in the HDL particles’ cholesterol content may impair HDL’s function.

ApoAI is the major protein and associated with intact HDL function [57,58]. Dysfunctional HDL contributes to the pathogenesis of atherosclerosis and cardiovascular diseases [59,60]. The oxidation of apoAI impairs the ABCA1-dependent activity [61]. In contrast, apoAI oxidation promotes amyloid formation [62] and inflammation [63]. In this study, apoAI oxidation was found and significantly increased in AD-HDL, particularly on the methionine residues 136 and 172 (Figure 4A,B). These findings were similar to the observation in patients with higher risks of cardiovascular diseases [64,65,66]. However, by using our UPLC/MS^E^ system, we did not identify tryptophan oxidation at position 72 as reported by the research mentioned above.

Previously, Kowal et al. showed that apoCIII could inhibit lipoprotein lipase and hepatic lipase, leading to the impairment of triglyceride metabolism [67]. Other than that, apoCIII induces the expression of vascular cell adhesion molecule 1 (VCAM-1), indicating its role in inflammatory responses [68]. Higher levels of apoCIII in HDL particles are associated with diabetes, dyslipidemia, atherosclerosis and coronary heart disease [69,70,71]. In this research, we also demonstrated that apoCIII was abundant in HDL from AD patients, primarily increased in the H4 and H5 subfractions (Figure 1D). Furthermore, while treating AD-H5 HDL to RAW 264.7 macrophages, it induced higher expression levels of GM1, phosphor-P65 and TNF-α (Figure 3B–D). As apoCIII was co-localized with GM1, apoCIII-rich AD HDL may be a possible mechanism of inflammation-mediated blood-brain barrier (BBB) disruption.

HDL functions are characterized by different chemical components, structures and electronegativity [36,50,72,73]. ApoE is a critical genetic risk factor for the development of AD, particularly apoE4, which changes one functional group of amino acids, increases the risk 4–15-fold and plays a role in the pathogenesis of Aβ deposition and neuroinflammation [74]. More recently, we showed that the glycosylation of apoE altered the hydrophobicity and receptor selectivity, leading to the impairment of lipid metabolism [42]. Here, we identified oxidation in apoE of AD-HDL in the H3–H5 subfractions of control-HDL and all fractions of AD-HDL (Figure 4C–E).

## 5. Conclusions

In conclusion, AD patients were shown to have increased electronegativity of HDL. The functional protein contents albumin was reduced; in contrast, the pro-inflammatory components such as apoCIII, and the oxidation of apoAI and apoE were enhanced in AD-HDL. In addition, the protein to lipid ratio was also decreased in AD-HDL. These findings support the potential role of AD-HDL involved in Aβ assembly and neural inflammation, which remain to be tested. The compromised cholesterol-efflux capacity of AD-HDL might furthermore contribute to cognitive impairment.

## Figures and Tables

**Table 1:** Biochemical profiles of the healthy controls and patients with Alzheimer’s disease.

	Controls (*n* = 24) Mean ± SD/Media (IQR)	AD (*n* = 44) Mean ± SD/Media(IQR)	*P*-Value of Two-Sample t-test /Wilcoxon Rank-Sum
Gender, M:F	5:19	10:34	0.8572#
Age, y	50.46 ± 10.34/51.00 (15.50)	79.69 ± 8.94/80.35 (11.00)	< 0001****/< 0001****
GPT (U/l)	22.33 ± 11.90/20.50 (11.00)	17.88 ± 7.45/16.00 (7.00)	0.1074/0.1863
T-CHOL (mg/dL)	208.9 ± 32.6/205.0 (58.5)	178.3 ± 43.0/175.0 (67.0)	0.0037*/0.0048**
TG (mg/dL)	121.3 ± 52.5/108.5 (78.5)	119.1 ± 73.7/99.5 (70.5)	0.9021/0.4333
HDL-C (mg/dL)	57.00 ± 11.75/56.00 (19.00)	48.31 ± 13.44/47.20 (17.90)	0.0107*/0.0094**
LDL-C (mg/dL)	124.1 ± 31.9/119.0 (42.5)	99.8 ± 32.0/99.3 (36.6)	0.0042*/0.0079**
HDL components			
Protein (%)	77.30 ± 5.15/76.18 (7.89)	68.70 ± 13.20/69.70 (24.96)	0.0003***/0.0198*
TG (%)	0.08 ± 0.03/0.07 (0.03)	0.21 ± 0.12/0.17 (0.10)	< 0001****/< 0001****
PL (%)	4.63 ± 1.05/4.77 (1.52)	5.77 ± 2.39/5.41 (4.22)	0.0083*/0.1101
FC (%)	5.69 ± 1.26/5.72 (1.56)	7.12 ± 3.14/7.42 (5.70)	0.0100*/0.1799
CE (%)	12.31 ± 3.27/12.87 (5.05)	18.2 ± 8.12/18.36 (15.56)	< 0001****/0.0072**

# Chi-squared test. P-value of two-sample t-test or Wilcoxon rank-sum: * *p* < 0.05, ** *p* < 0.01, *** *p* < 0.001, **** *p* < 0.0001, compared with controls and AD patients. Abbreviations: M: male; F: female; y: years; GPT: glutamic-pyruvate transaminase; T-CHOL: total cholesterol; TG: triglycerides; HDL-C: high-density lipoprotein-cholesterol; LDL-C: low-density lipoprotein-cholesterol; PL: phospholipids; FC: free cholesterol; CE, cholesteryl ester; SD: standard deviation; IDR: interquartile range; t-test: two-sample t-test).
